# Answer to Dr. Drake’s Interrogatories Continued

**Published:** 1853-04

**Authors:** 


*Answer to Dr. Drake's Interrogatories.—Continued.*

The question as it regards the early decay of the deciduous teeth—from the view of the subject to which we have drawn attention, presents first the constitutional cause or predisposition to disease, and second the exiciting cause of the disease itself. The first is an imperfect constitutional development. The second is the action of such agent or agents as may decompose their structure. The first is an inheritance from the parent. The second, may be the result of disease, vitiating the secretions which are continually brought in contact with the teeth—or the direct application of such agents to these organs as may decompose them.

However imperfectly developed these teeth may be, yet remove them from the mouth and keep them free from the action of corroding agents, and no decay takes place. In perfect health it would be contrary to the laws which govern the animal economy for one organ to destroy another. If we take a survey of those diseases to which children are so often subject, but seldom would we find the secretions of the mouth in a healthy condition. Take first the effect which teething itself generally produces. How often is the organ, for days before eruption, enveloped or at least covered by an irritable and inflamed mucous membrane? To this is added bowel complaints; the entire length of the alimentary canal is deranged; ulcers cover the mouth, the gums become inflamed—the secretions acrid and viscid, and tend to keep up the condition of things already described.

In investigating the causes of decay, the inquiry is a very natural one; What are the conditions of the secretions of the mouth during disease? Simon remarks, that he has "frequently seen the saliva acid in acute rheumatism, and in cases of salivation;" and "according to Donnè, the salvia has an acid reaction in all cases of irritation and inflammation of the stomach, in pleuritis, encephalitis, intermittent fevers, acute rheumatism, uterine affections, &c." The latter gentleman also remarks, that the secretions of the mucous membrane of the mouth is acid."* The next enquiry would be, What are the acids found in these secretions? That most frequently

*Harris's Principles and Practice of Dental Surgery. page 255.

found is perhaps the lactic. But very often the acetic is also present. The oxalic, nitrous, and others, have also been detected. Are then these acids injurious to the teeth? To what extent the first may be injurious we cannot positively speak, but the remainder are known to be highly destructive, the most of them destroying the teeth with great rapidity, and from the frequency with which some of these (particularly the acetic, and some other acids not detected in the saliva,) are brought in contact with the teeth, it is a matter of some surprise that these organs are not more universally destroyed by them. Donnê remarks that acidity of the saliva is found in patients affected with "gastritis and in children with aphthæ." The second summer for children we believe is generally regarded as one of peculiar danger, and why? Because they are then teething, and suffer most from bowel complaints, aphthæ, &c.

The decay of the deciduous teeth will then to a great extent depend on their constitutional development, and the extent to which the child has suffered from disease.

The view which we have here presented, we are satisfied will be verified by the closest observation. Children's teeth decay more prematurely than adults only so far as they are less perfectly organized, and suffer more from the causes to which we have alluded. Their natural position never being crowded, predisposes them to less decay than the adult. Their general want of care and their almost universal diet of sweet meats cannot but be prejudicial, if not to the teeth at least to the general constitution. There are many of the candies, sour drops, &c., daily used by females and children, which contain acids highly destructive to the dental organs. From the experiments of Prof. Westcott, "Raisins so corroded the enamel in twenty four hours, that its surface presented the appearance and was of the consistency of chalk." The decay making its appearance in the deciduous molar teeth, is found in the same localities, in the indentations and on the approximal surfaces just as in the adult. We also find the front teeth in both sets decay very similarly, the disease being most frequently seated on the approximal surfaces. The only difference might be traced to the neglect of the brush, which prevents decay from seating itself on the labial face of the teeth when used, and the more frequent derangement of the mucus secretions in the child. These scretions remaining about the teeth from day to day, decompose their structure.

Children who early suffer much with disease of the primæ viæ, have as a matter of course vitiated secretions, and anti-acid preparations so frequently used by practicing physicians in such diseases, show that this acrid condition is expected to be overcome at least in part by neutralizing agents, so that as these secretions pass through the alimentary canal, they may not keep up a continued irritation. While this condition remains we see irritable gums and their free margins surrround-ing the necks of the teeth, become red and much inflamed, and continually throw out an acid condition of the secretions from the mucous membrane.

The secretions thus thrown out, must, if left on the teeth, induce decay. It might be asked, why are not all the teeth alike affected from this cause? We remark because the fresh saliva is not alike thrown around them all, and hence those longest retaining the acrid secretions suffer most, and hence the front teeth below suffer less, and the upper most. The latter, during the sleeping moments of the child, may for hours be moistened with little else than the secretions from the mucous membrane. Delabarre a French writer of great observation remarks, when treating on dentition and the diseases of childhood, that "in studying the disease, to which children are the most disposed, we find that those which affect the mucous membrane hold the first rank; now, ought the physician, whose judgment should never be uncertain, who does not forget to question the patient that he sees for the first time, and thus inform himself on the temperament of the subject, neglect to examine the teeth where is found, written in indelible characters, the kind of affection to which the subject was disposed in childhood, and which must always, or at least for a *long time*, *have a great tendency to appear* again.

Your second question, which indeed embraces several interrogatories, we will consider somewhat separately. The first two being most intimately connected with the one already discussed.

You ask, "To what causes, external or pathological, local or constitutional, shall we ascribe the premature decay of the second teeth in the West?" and, "Is a hereditary, scrofulous diatheses a cause of infirm teeth?"

In the development of the teeth, that particular form of constitution, which would affect injuriously, the constitutional organization of the deciduous organ, would also act similarly on the teeth of replacement. The organs engaged in the process of assimilation, are alike engaged in the production of the same kind of an organ, and the rudiments of the permanent set are produced before birth, and up to this period, at least, they are alike affected by the constitutional vigor of the mother. This condition lasts until the end of lactation, when the child derives its nourishment from another source than the mother. We have already alluded to the different quality of that nourishment afforded by the mother, and the causes operating to deteriorate the elements for the tooth's production.

We have also alluded to the effect of diet and disease, on this operation of the animal economy, and as we think, explained to some extent, satisfactorily, the cause of a defective dental organization. In further confirmation of the constitutional and pathological causes inducing decay of the dental organs, we refer to our previous remark, which was—that "in lymphatic temperaments, with a scrofulous diathesis, where derangement of the mucous membrane so often terminates in phthisis pulmonalis, frail teeth are a general accompaniment." We have here, an enfeebled mucous tissue, and however perfect and symmetrical in form, the teeth may appear, yet, they early show symptoms of imperfect organization, possessing, it is true, an extraordinary amount of sensibility, yet the earthy particles of the dentine and enamel are not sufficiently compact to resist the chemical action of the itiated state of the secretions, furnished by this state of the mucous membrane.

This class of teeth presents to the dentist, more difficulty, in their preservation, than any other. We have a more than ordinary tendency of the organ to decay. This is accompanied, almost always, by excessive tenderness of the teeth—so much so, that the removal of the caries gives excruciating pain, and unless this irritable or inflamed condition of the dentine is subdued, and the secretions kept healthy, decay will progress. It is not unusual in these cases, to find the Incisors decaying, before the Bicuspides make their appearance.

In cases where we find the constitutional health improving, about the first thing to be noticed, is a lessened tendency to decay of the teeth, with diminished sensibility of the dentine. We have now under observation, quite a number of cases where the constitutional health is much improved, and the teeth not more than ordinarily sensitive. The dentine has become hard and glassy, and the teeth have lost their pearl-like hue. We could state numerous cases, very numerous indeed, where the teeth and constitution both gave way; medical and dental treatment, were both unavailing.

We think, from much observation, that dental irritation exerts a far more pernicious influence on the general health, than is supposed. Almost any dentist of much experience, could cite many cases, where, after the removal of all cause of dental irritation, the general health becomes much improved, and indeed, life evidently prolonged.

We regard then, an "hereditary, scrofulous diathesis, a cause of infirm teeth." We shall now consider some of the local or external causes, inducing decay of the teeth.

At first view, it appears strange that the most hard and dense part of our organization, is so often the first to yield to the ravages of disease, and if we apply here, the same theory, as it regards the cause of decay, as is applicable to other bones, we should find ourselves at fault; for inflammation and rottenness are more frequent in the soft bones, and the harder portion, even of the same bone, is less liable to be affected. The teeth being so much more dense than any of the other bones, would, from the same causes, scarcely ever decay. We must, therefore, look to some other cause of decay, than that which operates on the bones of the general system. That portion of the teeth in which disease generally commences, is situated differently from any of the other bones—they are not covered by the soft parts. The crown of the tooth, instead of being covered with a periostrum, is covered with an enamel, a hard and crystalized substance, possessing but little if any vitality. Close observation will almost invariably detect a disintegration of the crystals which protect the bony structure. This disintegration, (or decomposition, as is most frequently the case,) always precedes caries of the bone beneath; the latter being softer, decomposes much more readily, and hence, although the opening in the enamel may be small, yet the destruction of the bone beneath, may be found very extensive.

The question then comes up, what is the primary local cause of the disease. If we consider the constituents of the tooth itself, and the nature of its organization, we will find that an agent capable of breaking up this combination of elements, must have a greater affinity to some one of the elements than that which holds them in combination. Can and does this ever take place? We find the tooth's constituents to be phosphate of lime, with about one-third of animal matter, and small traces of other earthy particles. The phosphate of lime, however, and animal matter, constitute so near the entire particles, that to these alone must we look for that resistance to chemical agency which may be brought to bear on these organs. If we now take up the acids, we will find several having a greater affinity to the lime than the phosphoric. We have for instance the oxalic, sulphuric, succinic, and tartaric. Are then these acids ever brought in contact with the teeth. Two are very frequently. These are the tartaric and sulphuric. It may be remarked, that they are always diluted so much when taken in the mouth (and by the presence of saliva) that they cannot injure. But this is not the case. The natural temperature of the mouth very much increases the action of these acids, and these as well as the muriatic, nitric and citric, though largely diluted, act with considerable rapidity on the teeth. The lemon juice contains the citric acid strong enough to act injuriously. How much of the acetic acid is manufactured out of the sulphuric acid. We see evidences of this very often in the effect which such vinegar has upon pickles, &c. These are softened by its use and when taken into the mouth must injure the teeth. We have then the acidulated drops, now almost as common as candy. These are made sour, by, we believe, the citric or tartaric acid, and that the acid they contain, may have all chance to act on the teeth, they are merely held in the mouth until the heat and moisture dissolve them. We could warrant the destruction of the best set of teeth by such continued treatment. It is needless to enumerate cases where acids may be brought in contact with these organs. Some are used as tonics, some as ingredients in beverages, some are found in the mouth as the result of the decomposition of vegetable and animal matter about the teeth, some are contained in our articles of diet, and it would appear impossible to keep them from the teeth.

"Salts whose acids have a stronger affinity for the lime of the teeth, than for the basis with which they are combined, are decomposed, the acids acting upon the teeth."

The action of some of the acids appear to be directly on the earthy part of the dentine—while others act not only on this, but also on the animal portion, and we think the different varieties of decay may all be accounted for by the nature of the chemical agent decomposing the teeth and its constitutional organization. The increased and diminished sensibility of the teeth bone can also be thus accounted for. Sulphuric acid, for instance, would decompose the teeth rapidly and increase the sensibility of the dentine, producing inflammation to some extent in the bony structure, other agents acting more slowly deaden the vitality of the bone as fast as it acts upon it. The primary local cause of decay we think is some chemical agent, the action of which decomposes the tooth's substance.

The location of decay fully confirms this opinion, for almost invariably we find decay commencing in the deep indulations of the teeth and on their approximal surfaces, where the teeth press upon each other and retain such agents until they have time to act.

The mere rinsing the mouth with water after an acid has been used, will not immediately neutralize it.

The acid penetrates deeper into the indentations, and if there should be a crack in the enamel, it enters there, and is retained in such situations, and between the teeth until it unites with either the lime or animal portion, breaking up the union which exists in these elements as constituted in the tooth. The more smooth and perfect the enamel, the more hard and compact the dentine, the better will they resist the action of corrosive agents. Ivory as coarse as that in the tusk of the Hippopotamus will last much longer in the mouth by being well polished and the surface rendered hard by the action of the burnishing instrument. The cells or tubuli are thus closed and prevent the sudden ingress of the corroding agent.

On the same principle, teeth imperfectly organized cannot so well resist the action of such agency. And such are generally found in the mouths of those of infirm general constitutional health, and the secretions in which they are constantly bathed are more apt to be vitiated. Unless therefore the general constitution becomes changed for the better, the same cause which gives an imperfect dental organization, operates to destroy that which has already been produced.

